# Joint analysis of left ventricular expression and circulating plasma levels of Omentin after myocardial ischemia

**DOI:** 10.1186/s12933-017-0567-x

**Published:** 2017-07-07

**Authors:** Louis A. Saddic, Sarah M. Nicoloro, Olga T. Gupta, Michael P. Czech, Joshua Gorham, Stanton K. Shernan, Christine E. Seidman, Jon G. Seidman, Sary F. Aranki, Simon C. Body, Timothy P. Fitzgibbons, Jochen D. Muehlschlegel

**Affiliations:** 1 Department of Anesthesiology and Perioperative Medicine, University of California Los Angeles, Los Angeles, CA USA; 2 Program in Molecular Medicine, University of Massachusetts Medical Center, Worcester, MA USA; 3 UT Southwestern Medical Center, Dallas, TX USA; 4 Program in Molecular Medicine, University of Massachusetts Medical School, Worcester, MA USA; 5 Department of Genetics, Harvard Medical School, Boston, MA USA; 6 Department of Anesthesiology, Perioperative and Pain Medicine, Brigham and Women’s Hospital, Harvard Medical School, CWN L1, 75 Francis Street, Boston, MA 02115 USA; 7 Division of Cardiovascular Medicine, Brigham and Women’s Hospital, Howard Hughes Medical Institute, Boston, MA 02115 USA; 8 Division of Cardiac Surgery, Brigham and Women’s Hospital, Harvard Medical School, Boston, MA USA; 9 Cardiovascular Division, Department of Medicine, University of Massachusetts Medical School, Worcester, MA USA

**Keywords:** Ischemia, Adipokine, Omentin, RNA-seq, Cardiovascular

## Abstract

**Background:**

Omentin-1, also known as Intelectin-1 (ITLN1), is an adipokine with plasma levels associated with diabetes, obesity, and coronary artery disease. Recent studies suggest that ITLN1 can mitigate myocardial ischemic injury but the expression of *ITLN1* in the heart itself has not been well characterized. The purpose of this study is to discern the relationship between the expression pattern of *ITLN1* RNA in the human heart and the level of circulating ITLN1 protein in plasma from the same patients following myocardial ischemia.

**Methods:**

A large cohort of patients (n = 140) undergoing elective cardiac surgery for aortic valve replacement were enrolled in this study. Plasma and left ventricular biopsy samples were taken at the beginning of cardiopulmonary bypass and after an average of 82 min of ischemic cross clamp time. The localization of ITLN1 in epicardial adipose tissue (EAT) was also further characterized with immunoassays and cell fate transition studies.

**Results:**

mRNA expression of *ITLN1* decreases in left ventricular tissue after acute ischemia in human patients (mean difference 280.48, *p* = 0.001) whereas plasma protein levels of ITLN1 increase (mean difference 5.24, *p* < 0.001). Immunohistochemistry localized ITLN1 to the mesothelium or visceral pericardium of EAT. Epithelial to mesenchymal transition in mesothelial cells leads to a downregulation of *ITLN1* expression.

**Conclusions:**

Myocardial injury leads to a decrease in *ITLN1* expression in the heart and a corresponding increase in plasma levels. These changes may in part be due to an epithelial to mesenchymal transition of the cells that express *ITLN1* following ischemia.

*Trial Registration* Clinicaltrials.gov ID: NCT00985049

**Electronic supplementary material:**

The online version of this article (doi:10.1186/s12933-017-0567-x) contains supplementary material, which is available to authorized users.

## Introduction

Adipose tissue is a complex organ whose functions extend well beyond its canonical role in energy storage [1–3]. Adipose tissue can act as an endocrine organ through the secretion of adipokines which have the ability to act locally and remotely. Adipokines have diverse roles including the regulation of angiogenesis, metabolism, inflammation, and cell survival/death in many tissue types. Recently, investigators have begun to uncover the pivotal role these secreted substances have in many pathological processes including cardiovascular disease [4–6].

Omentin-1, also know as Intelectin-1 (ITLN1), is an adipokine that in humans is primarily expressed in visceral adipose tissue (VAT), with very little expression in subcutaneous adipose tissue (SAT) [7]. More specifically, the vast majority of *ITLN1* expression from adipose tissue is derived from the stromal vascular compartment with only trace amounts produced in adipocytes [8]. This is in contrast to many other adipokines which are primarily expressed in fat cells [7]. In humans, ITLN1 can be detected in the circulation, and the level of this circulating pool of protein has been correlated with many disease processes. For example, the plasma concentration of ITLN1 is reduced in patients with obesity and diabetes [9, 10]. With respect to cardiovascular disease, ITLN1 is decreased in metabolic syndrome patients with carotid atherosclerosis [11] along with patients with coronary artery disease (CAD) [12, 13]. Functionally, ITLN1 has been shown to reduce TNF-induced vascular inflammation in human endothelial cells [14]. In rats, ITLN1 promotes vasodilation in blood vessels [15] and protects against cerebral ischemia by promoting angiogenesis and inhibiting apoptosis through stimulating AKT and endothelial nitric oxide synthase [16]. A recent study also showed that systemic administration of human ITLN1 in mice leads to a reduction in infarct size and myocyte apoptosis after ischemia/reperfusion injury through phosphorylation of AMPK and AKT [17]. These protective properties of ITLN1 are further substantiated by other studies which have shown that increasing levels of plasma ITLN1 in humans correlate with higher myocardial index scores [17] and a lower frequency of ischemic EKG changes [18].

Despite these preliminary studies, the expression of this cardioprotective adipokine in the heart has not been well characterized. Studies have shown that the epicardial fat, a special type of visceral fat, expresses high amounts of ITLN1 and this expression may be related to underlying CAD [19, 20]. Early studies on the global expression of ITLN1 demonstrated that this adipokine has low levels of expression in other organs including the heart [7] but further studies in humans have been limited given the difficulty of obtaining human heart tissue. Therefore, we sought to determine the effects of myocardial ischemia on the expression of *ITLN1* in the human left ventricle and circulating levels of ITLN1 protein in a large population of patients.

## Methods

### Human subjects and left ventricular samples

Patients presenting for elective aortic valve replacement surgery by a single surgeon were enrolled in this study. They provided written informed consent with Institutional Review Board approval. Clinical characteristics of the study population are shown in Table 1. Patients were deemed to have CAD if obstructive lesions were noted on left heart catheterization just prior to aortic valve surgery requiring concomitant revascularization of at least one vessel. Punch biopsies (~3–5 μg total RNA content) were obtained intra-operatively from the site of a routinely placed surgical vent in the anterolateral apical left ventricle at the initiation of cardiopulmonary bypass (CPB) and again after an average of 81.73 ± 27.89 min of aortic cross clamp time. Specifically, the biopsies were taken from within the myocardium and did not include infarcted tissue. Pre-ischemia and post-ischemia biopsies were taken from the same site in the track made for the LV vent. During aortic cross clamping, the heart was infused with intermittent cold blood cardioplegia (8:1 blood to crystalloid ratio) for myocardial protection.

**Table 1:** Clinical characteristics of study patients

Characteristics	Statistic max n = 140
Gender (male)	86 (61.43%)
Age	71.24 ± 12.09
Degree of aortic stenosis	
None to trivial	10 (7.14%)
Mild	0 (0%)
Moderate	15 (10.71%)
Severe	115 (82.14%)
Degree of aortic insufficiency	
None to trivial	61 (43.57%)
Mild	38 (27.14%)
Moderate	25 (17.86%)
Severe	16 (11.43%)
LV EF (%)	57.36 ± 10.97
AX time (min)	81.73 ± 27.89
BMI (kg/m^2^)	30.76 ± 7.13
CAD (%)	67 (47.86%)
HgA1c (%)	6.08 ± 0.90
Day 1 CKMB (mcg/L)	32.00 ± 24.57
Pre *ITLN1* (FPKM)	59.96 IQR 5.43–303.57 n = 132
Post *ITLN1* (FPKM)	2.46 IQR 0.84–11.00 n = 132
Pre ITLN1 (ng/ml)	32.23 ± 15.22 n = 106
Post ITLN1 (ng/ml)	37.46 ± 16.84 n = 106

Statistics are mean values and standard deviations for continuous variables except *ITLN1* mRNA levels which are described with median and interquartile range. Statistics for categorical variables are number of patients and percentage of patients

*LV EF* left ventricular ejection fraction, *AX time* aortic cross clamp time, *BMI* body mass index, *CAD* coronary artery disease, *HgA1c* hemoglobin A1c, *CKMB* creatine kinase MB fraction, *IQR* interquartile range

### RNA sequencing

Tissue samples were immediately placed in RNAlater^®^ (Ambion, Life Technologies, USA), and after 48 h at +4 °C were stored at −80 °C until RNA extraction. Total RNA was isolated with Trizol and RNA quality was assessed using the Agilent Bioanalyzer 2100 (Agilent) with no samples being excluded for poor quality. Library preparation and sequencing has been described previously [21], but briefly, ribosomal RNA was removed by performing 1–2 washings of RNA annealed to poly-T oligos beads (Invitrogen). RNAs were reverse transcribed using random hexamers (Invitrogen). Double-stranded DNA (dsDNA) synthesis was performed using Pol I and RNA-ase H. Short fragments were purified with QiaQuick PCR extraction kit (Qiagen) and resolved with EB buffer for end reparation and poly(A) addition then ligated with sequencing adaptors for cluster generation and sequencing on the Illumina HiSeq 2000 (Illumina, San Diego, CA). As samples were analyzed at different times, different read lengths were employed, initially using single-end reads and then transitioning to paired-end reads ranging from 36 to 100 base pairs.

### Read alignment, transcript quantification, and differential gene expression analysis

Raw reads produced by the Illumina sequencer imaging files were filtered to remove reads containing adaptor sequences, containing >5% unknown nucleotides, or having >50% of reads with base quality scores <5. Using Tophat v2.0.5 and Bowtie2 [22–24], reads were aligned to the Homo Sapiens reference genome (UCSC hg19). For paired-end reads, mate inner distance was set at 165 bp and mate standard deviation at 37 bp. For other parameters, we used the default settings in Tophat v2.0.5 and Bowtie2. Final read alignments having more than two mismatches or more than two gaps or more than two edit distances were discarded. The anchor length was 8 without any mismatches being tolerated. The minimum and maximum intron lengths were 70 and 500,000 respectively. The maximum insertion and deletion lengths were 3. Fragments per kilobase per million mapped reads (FPKM) levels were determined using Cufflinks [25]. Differential expression analysis was performed using Limma (v3.30.11) [26]. A linear model was constructed to test the effect of ischemia on gene expression, denoted y, while blocking on the patient from which each sample was collected, which adjusts for within and between-sample variability:

$${\text{y }}\sim {{\upbeta }}_{1} \left( {\text{patient}} \right) + {{\upbeta }}_{2} \,({\text{ischemia}})$$

where β denotes regression coefficients. Read counts were normalized while incorporating sample-level weights using voom [27], and genes were filtered for inclusion in differential expression analysis in accordance with practices established by the authors of edgeR [28]. For each gene, positive or negative log2fold change, which was interpreted as an increase or decrease, respectively, in gene expression due to ischemia, was determined. *p*-values were adjusted for multiple comparison testing using the Benjamini Hochberg method.

### Epicardial, visceral, and subcutaneous fat samples

Human visceral (omental) and abdominal subcutaneous adipose tissue samples were collected from patients undergoing weight loss reduction surgery at the University of Massachusetts Medical School between 2005 and 2009 [29]. The microarray data from visceral (VAT) and subcutaneous adipose tissue (SAT) samples have been deposited in the GEO database under accession code GSE20950. Samples of epicardial adipose tissue (EAT) adjacent to the right coronary artery, and SAT from the sternum, were collected from patients without CAD who were having elective surgery for valvular heart disease. RNA was isolated using QIAGEN mini-lipid RNA extraction kits and analyzed on an Agilent 2100 Bioanalyzer. Complementary RNA (cRNA) synthesis and hybridization to Affymetrix 1.0 ST microarrays was performed by the UMASS Medical School Genomics Core Facility. For both studies, informed consent was given by the patients and the study was approved by the University of Massachusetts Medical School Institutional Review Board. Subcutaneous (inguinal) and visceral (gonadal) adipose tissue was harvested from 13 week old male C57BL/6 mice fed normal chow. RNA from paired samples (n = 6) was prepared with QIAGEN mini-lipid RNA extraction kits and analyzed on an Agilent 2100 Bioanalyzer. Complementary RNA (cRNA) synthesis and hybridization to Affymetrix Mouse Gene 1.0 ST microarrays was performed by the UMASS Medical School Genomics Core Facility. This data is publically available in the GEO database under accession code GSE28440.

### ITLN1 ELISA

Plasma samples were obtained from venous blood obtained before and after CPB in the same population of cardiac surgical patients from which the left ventricular biopsies were taken. Concentrations of ITLN1 were measured using ELISA (Millipore #EZH0MNTN1-29K) according to the manufacturer’s instructions.

### ITLN1 Immunohistochemistry

Sections of EAT were fixed overnight in 10% formalin, sectioned, and stained for ITLN1 using a sheep anti-human antibody (R&D Biosystems #AF4254) at 10 μg/ml. The secondary antibody was a donkey Anti-Sheep IgG NorthernLights NL557-conjugated antibody. Sectioning and immunofixation were done by the UMASS Morphology core.

### Mesothelial cell culture and qRT-PCR

Primary human mesothelial cells were purchased from Zen-Bio. Cells were cultured according to the manufacturer’s instructions (Human Adult Mesothelial Cell Manual ZBM0025.01). Briefly, cells were thawed and plated in MSO-1 media at a density of 5000 cells/cm^2^. After they were 85–90% confluent, they were split at a 1:6 ratio and seeded onto collagen coated plates. On day 4, cells were serum starved for 24 h with Media 199 (Gibco) containing 5% penicillin/streptomycin, normocin, and 0.5% FBS. After 24 h, the media was changed and vehicle or 5 ng/ml TGFβ1(Sigma T7039) was added. Cells were harvested for RNA extraction after 48 h of TGFβ1 treatment. RNA was isolated using TRIzol as previously described. cDNA was synthesized with the iScript cDNA systhesis kit (BioRad) with 1 μg RNA template. qRT-PCR was performed using the iTaq Universal Sybergreen supermix (BioRad) and a CFX96 Connect Real Time Detection System. Relative expression was normalized to ribosomal phosphoprotein P0 (RPLP0) using the method of Livak et al. [30]. Primer sequences were obtained from primer bank and were as follows: E-cadherin (*CDH*-*1*) (5′cgagagctacacgttcacgg 3′, 3′gggtgtcgagggaaaaatagg 5′); *VMAC* (5′gccctagacgaactgggtc 3′, 3′ggctgcaactgcctaatgag5′); fibronectin (5′cggtggctgtcagtcaaag3′, 3′aaacctcggcttcctccataa5′); *SNAI1* (5′ tcggaagcctaactacagcga 3′, 3′agatgagcattggcagcgag 5′); *SNAI2* (5′cgaactggacacacatacagtg 3′, 3′ctgaggatctctggttgtggt 5′).

### Statistical analysis

Baseline continuous variable patient characteristics are presented as means and standard deviations except *ITLN1* mRNA levels which are described with median and interquartile range. Categorical variables are described with total number and percentages. Differences between pre-ischemia and post-ischemia ITLN1 protein levels and *ITLN1* RNA levels were determined using a paired *t* test. Univariate linear regression analysis was performed on pre-ischemia *ITLN1* RNA and ITLN1 plasma levels against each other and clinical parameters including age, body mass index (BMI), hemoglobin A1c (HgA1c), sex, CAD, and post-operative day 1 creatine kinase MB fraction (CKMB). Similarly, univariate linear regression analysis was also conducted on post-ischemia *ITLN1* RNA and ITLN1 plasma levels against the same clinical parameters in addition to aortic cross clamp time. These studies were conducted in R studio [31] using the general linear model function and results are reported as *p*-values and beta coefficients. *p*-value <0.05 was considered significant.

## Results

### Plasma ITLN1 protein levels increase while left ventricular ITLN1 RNA expression decreases following myocardial ischemia in humans

**Fig. 1:**
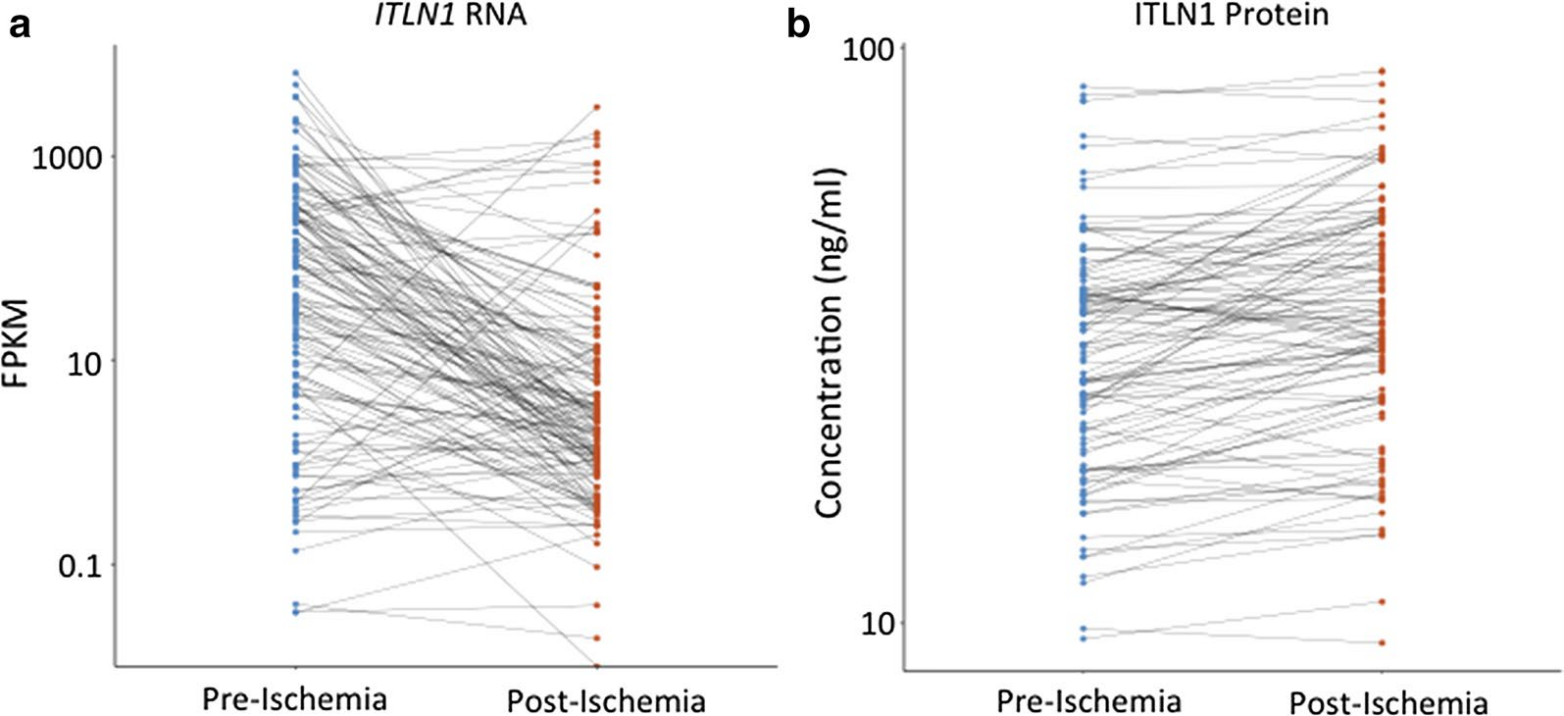
*ITLN1* expression is decreased in the left ventricle and circulating plasma levels of ITLN1 are increased following acute ischemia in the human heart. Stripcharts display the change in *ITLN1* expression in left ventricular tissue samples (**a**) and in circulating ITLN1 protein levels following acute ischemia in the heart (**b**). FPKM: fragments per kilobase per million mapped reads

We used our prior CPB model in patients undergoing aortic valve replacement to obtain pre-ischemia (blood and left ventricular tissue harvested at the start of CPB) and post-ischemia samples (tissue harvested after a mean of 81.73 ± 27.89 min of ischemic cross clamp time) [21]. This model has previously been validated to mimic ischemia in human hearts [32, 33]. In a small cohort of patients, *ITLN1* was the most significantly down-regulated gene in this model [21]. In the current larger study from 132 paired samples, median levels of pre-ischemia and post-ischemia left ventricular *ITLN1* RNA expression were 59.96 FPKM (interquartile range 5.43–303.57) and 2.46 FPKM (interquartile range 0.84–11.00) respectively. In this population, post-ischemia RNA levels were significantly lower than pre-ischemia levels (mean difference 280.48, 95% CI 449.43–111.54, *p* = 0.001) (Fig. 1a). From the same group of patients, 106 paired samples had mean pre-ischemia and post-ischemia ITLN1 plasma levels of 32.23 ± 15.22 and 37.46 ± 16.84 ng/ml respectively. In this population post-ischemia protein levels were significantly higher than pre-ischemia levels (mean difference 5.24, 95% CI 3.52–6.95, *p* < 0.001) (Fig. 1b). In patients with CAD compared to those without, pre-ischemia left ventricular expression levels of *ITLN1* were slightly higher (median 89.64 FPKM interquartile range 4.60–331.00 vs median 43.53 interquartile range 5.62–281.85, *p* = 0.839) while pre-ischemia plasma levels of ITLN1 were slightly lower (mean 30.94 ± 14.44 ng/ml vs mean 33.56 ± 16.03 ng/ml, *p* = 0.378) but these associations were not significant.

We next investigated the correlation between pre-ischemia and post-ischemia *ITLN1* expression in the left ventricle and clinical parameters using univariate linear regression models. There were no significant correlations identified. We repeated these correlations with plasma protein levels. Age was positively correlated with plasma protein levels before and after ischemia (Table 2). There were no significant associations of plasma protein levels or left ventricular expression of *ITLN1* with patients on or off statin medications (data not shown).

**Table 2:** Univariate correlations between *ITLN1* RNA expression and circulating levels of ITLN1 protein with clinical parameters

Characteristic	Beta	*p*-value
Pre-ischemia *ITLN1* RNA		
Age	1.586	0.81
BMI	−0.40	0.971
HgA1c	−1.05	0.99
Sex (male)	−18.42	0.91
CAD	32.15	0.839
Day 1 CKMB	1.80	0.582
ITLN1 pre-ischemia protein	−1.06	0.879
Post-ischemia *ITLN1* RNA		
Age	1.08	0.683
BMI	−0.43	0.923
HgA1c	−29.44	0.404
Sex (male)	−4.55	0.944
CAD	−2.97	0.962
Day 1 CKMB	−0.39	0.756
AX time	−0.50	0.660
Pre-ischemia ITLN1 protein		
Age	0.30	0.022*
BMI	−0.20	0.340
HgA1c	2.18	0.225
Sex (male)	3.37	0.260
CAD	−2.36	0.424
Day 1 CKMB	−0.01	0.900
*ITLN1* pre-ischemia RNA	−0.0002	0.879
Post-ischemia ITLN1 protein		
Age	0.37	0.011*
BMI	−0.26	0.250
HgA1c	1.64	0.410
Sex (male)	−1.00	0.765
CAD	−0.24	0.942
Day 1 CKMB	−0.04	0.524
AX time	−0.01	0.862

*BMI* body mass index, *HgA1c* hemoglobin A1c, *CAD* coronary artery disease, *CKMB* creatine kinase MB fraction, *AX time* aortic cross clamp time

* Denotes significance

### Epicardial and visceral adipose tissue have a similar gene expression profile, characterized by enrichment of genes encoding proteins involved in innate immunity

**Fig. 2:**
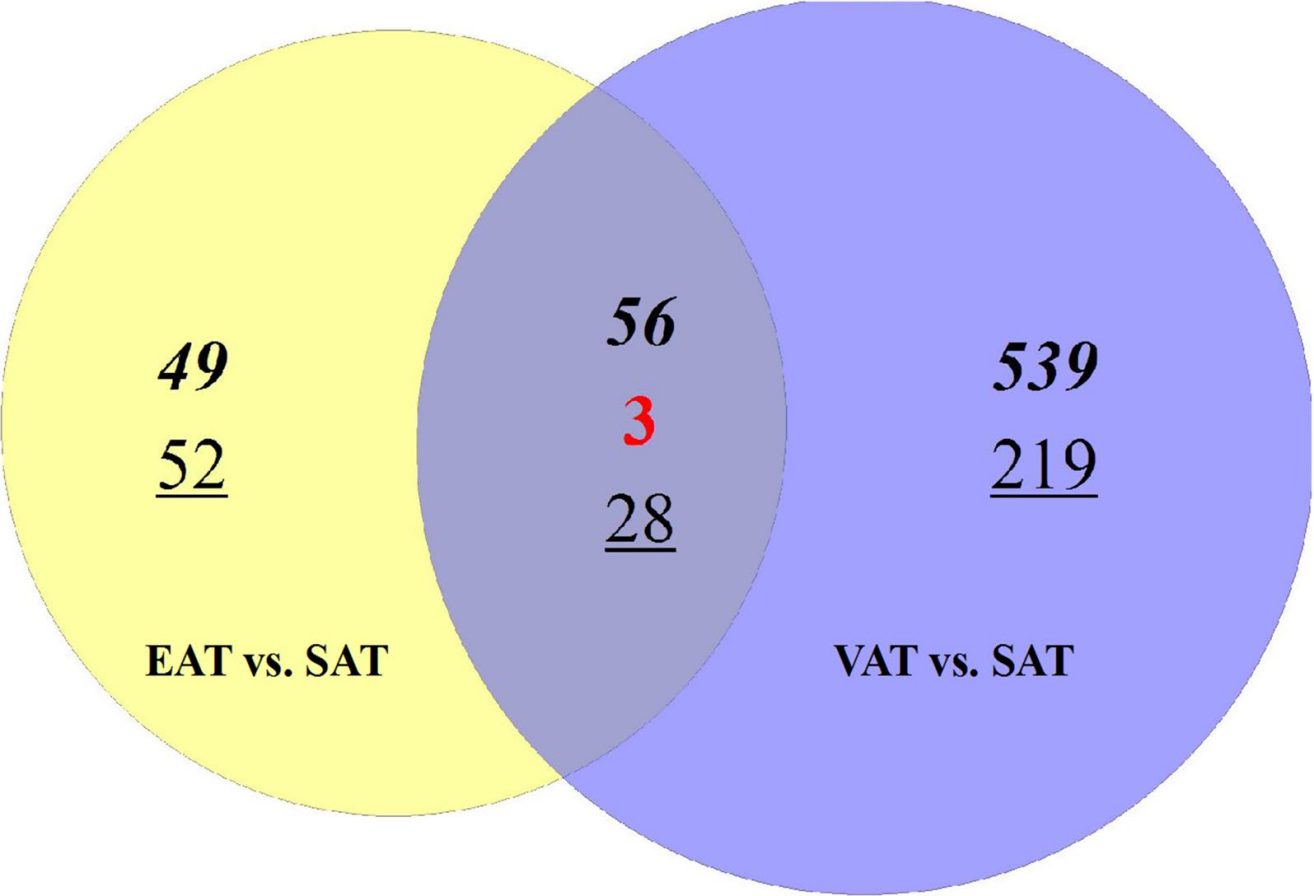
Venn diagram of differentially regulated genes between subcutaneous adipose tissue (SAT) vs. visceral adipose tissue (VAT) and epicardial adipose tissue (EAT) vs. SAT. In the SAT vs. VAT comparison, 845 genes were differentially regulated (FC > 2.0, *p* < 0.05). In the EAT vs. SAT comparison 188 genes were differentially regulated. 46% of these were commonly differentially regulated in VAT in comparison to SAT (middle overlapping segment). The *numbers* in *each circle* indicate the number of genes whose expression was altered; *numbers* in *bold italic* are upregulated, those in *red font* are contraregulated, and those in *underlined plain font* are downregulated in the respective comparisons. The specific genes are listed in Table 3

Given the proximity of our cardiac biopsy samples to epicardial adipose tissue (EAT), which has also been shown to express *ITLN1* [8, 19], we further wanted to characterize the expression of *ITLN1* in this compartment. Clinical studies have demonstrated that the amount of EAT correlates with visceral adipose tissue (VAT), and is increased in aging and obesity [34, 35]. Therefore we hypothesized that the transcriptome of EAT may be similar to that of VAT, including high expression of *ITLN1*. In order to compare the gene expression profile of VAT with EAT, we utilized our previously published work [29]. We first compared gene expression in paired SAT and VAT samples from eight non-diabetic patients who underwent weight reduction surgery. Next, we compared gene expression in paired EAT and SAT samples from nine patients without CAD having surgery for heart disease. We then evaluated the two sets of differentially regulated genes in order to indirectly compare the gene expression of EAT and VAT. In the paired VAT vs. SAT comparison, there was differential expression of 845 genes (Fold change >2.0, *p* < 0.05). In the paired SAT vs. EAT comparison, 188 genes were differentially expressed (FC > 2.0, *p* < 0.05). Of these 188 genes, 46% were commonly differentially regulated in VAT (Fig. 2). These included *ITLN1* and other canonical VAT specific genes such as *TCF21, SRFP2*, and *ALOX15* (Table 3). Remarkably, geneset enrichment analysis of the differentially expressed genes in both the SAT vs. VAT and SAT vs. EAT comparison yielded almost identical results (Table 4). The top three enriched pathways in VAT and EAT included complement, classical complement, and lectin induced complement. Therefore, even in the absence of CAD or diabetes, both VAT and EAT seem to play an important role in innate immunity.

**Table 3:** Genes whose expression are commonly regulated in visceral adipose tissue (VAT) and epicardial adipose tissue (EAT) in comparison to subcutaneous adipose tissue (SAT)

Commonly up regulated in VAT and EAT (n = 56)	Contra regulated in VAT and EAT (n = 3)	Commonly down regulated in VAT and EAT (n = 28)
*ITLN1,TGM1, PROX1, IL18, C3, FRAS1, PDPN, PLAT, UPK3B, PDE1A, CGNL1, OSR1, GATA6, KCNT2, C21orf62, FBLN1, DSC3, TFF3, RELN, SULF1, CFI, FLRT3, PLLP, SLC39A8, GLT8D4, BCHE, MEIS2, PTPRD, ALDH1A2, HSD17B6, GPM6A, ART4, TIMP1, DKFZP586H2123, SYT4, BNC1, MSLN, PTGDS, LRP2, TGM2, ITGB8, PTN, ALOX15, C4A//C4B, CLDN1, RARRES1, INMT, MMRN1, UPK1B, SLPI, C7, TCF21, CKMT1A, CCL21, HP, PKHD1L1,*	*SFRP2, CD36, CHI3L2*	*NNAT, C12orf39, CXCL14, CRHBP, XG, ALDOC, ABCD2, EGFL6, ADRA2A, MFAP5, CPM, APOB, SIM1, SIX1, NOVA1, TBX15, SNX10, ENPP1, MME, OSR2, CDKN2B, DGAT2, FOSB, CCND1, TNN, TBX5, ZFP36, KLB*

**Table 4:** Gene set enrichment analysis of pathways enriched in epicardial adipose tissue (EAT) and visceral adipose tissue (VAT) vs. subcutaneous adipose tissue (SAT)

	Enrichment (%)	*p*-value
Epicardial adipose tissue		
Classical complement pathway	28	<0.001
Lectin induced complement pathway	25	<0.001
Complement pathway	21	<0.001
Cell/molecules of local acute inflammatory response	17	<0.001
Visceral adipose tissue		
Classical complement pathway	28	<0.001
Complement pathway	26	<0.001
Lectin induced complement pathway	25	<0.001

### *ITLN1* expression is localized to the mesothelium and downregulated following epithelial to mesenchymal transition

**Fig. 3:**
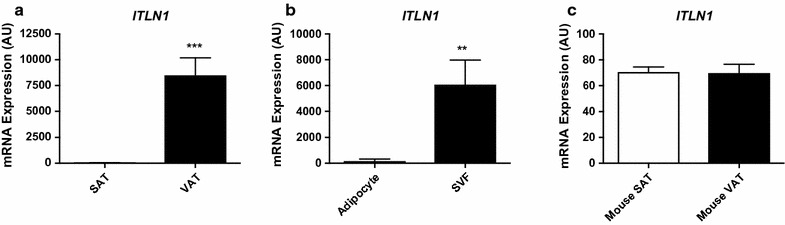
*ITLN1* is specifically expressed in the stromal vascular fraction of visceral fat in humans. **a** Microarrays were queried for expression of *ITLN1* in visceral adipose tissue (VAT) vs. subcutaneous adipose tissue (SAT) in paired samples (n = 8) (*** *p* < 0.001). **b** *ITLN1* expression from microarrays of the adipocyte and stromal vascular fraction (SVF) of VAT in paired samples (n = 6) (** *p* < 0.01). **c** Expression of *ITLN1* from microarrays of murine SAT and VAT

Next, we aimed to more carefully characterize the expression of *ITLN1* in EAT. We first confirmed that expression of *ITLN1* is highly enriched in human VAT, and nearly absent in SAT (Fig. 3a). Similar to other studies [7, 8], we also were able to show that *ITLN1* expression in human VAT is isolated to the stromal vascular fraction (SVF) (Fig. 3b). Mice, on the other hand, have equivalent low levels of *ITLN1* expression in both VAT and SAT (Fig. 3c).

**Fig. 4:**
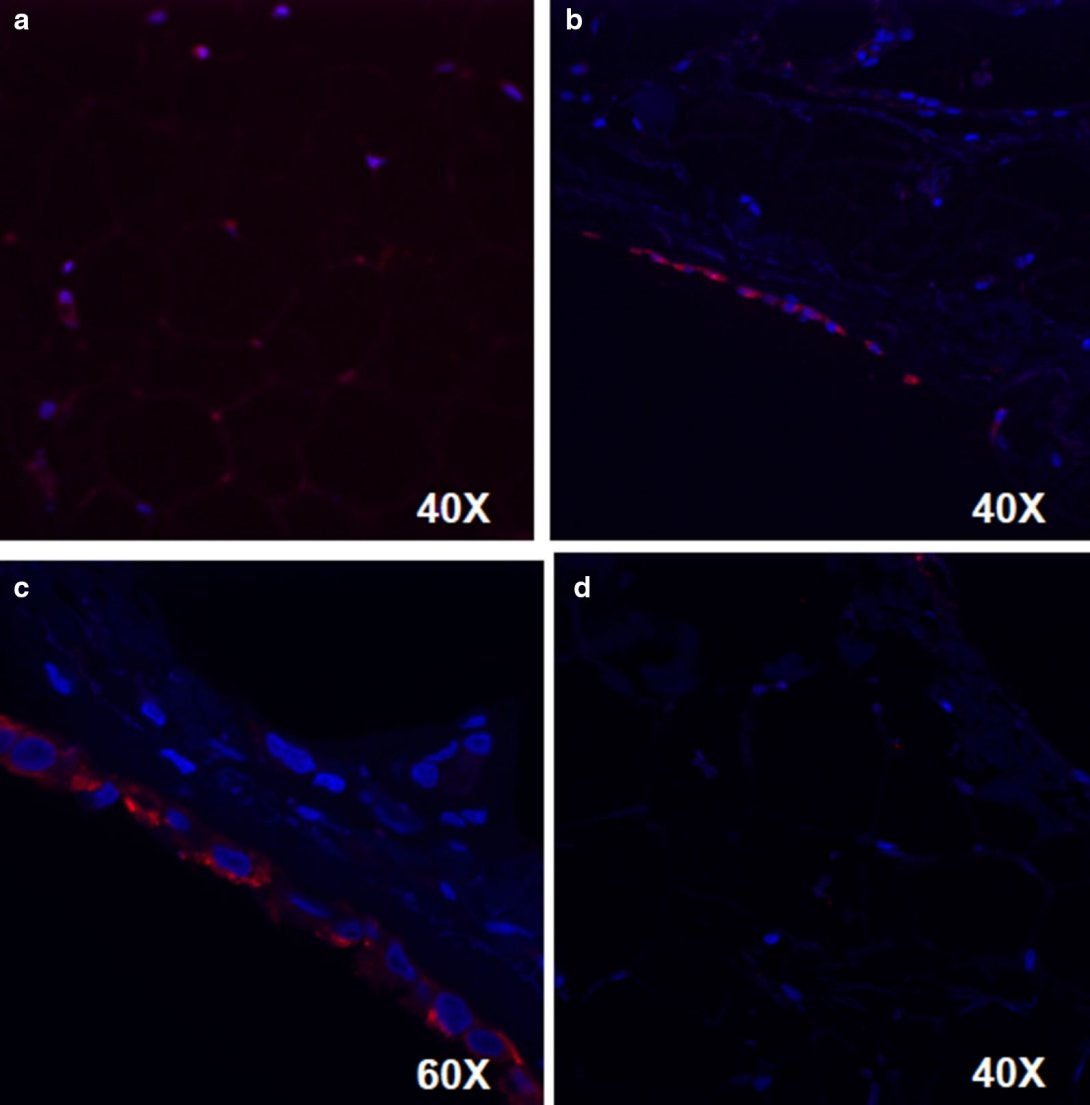
Immunohistochemistry of ITLN1 in epicardial adipose tissue (EAT) shows enrichment within the mesothelial cell layer. Subcutaneous adipose tissue (SAT) stained for ITLN1 (**a**). EAT stained for ITLN1 (**b**) with magnification of the mesothelial cell layer of the visceral pericardium (**c**). Secondary antibody alone was used as a control (**d**). ITLN1* red*, DAPI* blue*

Since the SVF can contain multiple different cell types including epithelial cells, macrophages, fibroblasts, and precursory adipocytes [7], we sought to further characterize the location of ITLN1 in EAT. Immunohistochemistry demonstrated that ITLN1 is predominantly localized to the mesothelium, which constitutes the visceral pericardium, and not the adjacent adipocytes (Fig. 4a–d).

**Fig. 5:**
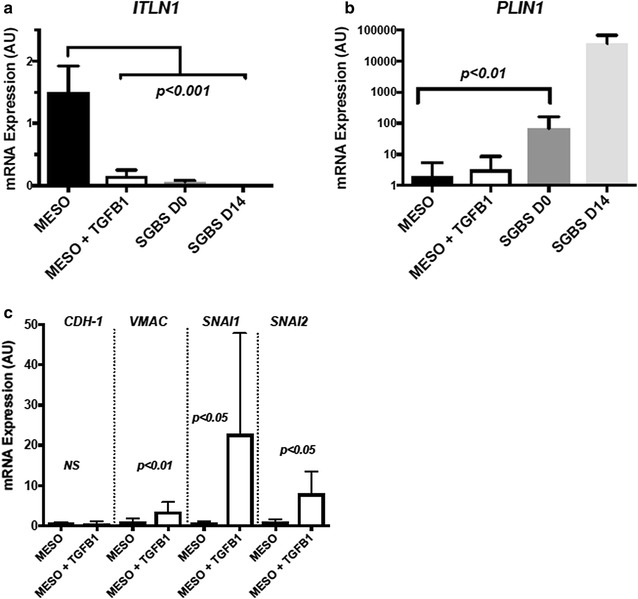
*ITLN1* expression in primary mesothelial cells is dramatically reduced following an epithelial to mesenchymal transition. **a** qRT-PCR for *ITLN1* in mesothelial cells ± TGFβ1 and in a human adipocyte cell line (SGBS) before (D0) and after differentiation (D14) (n = 6 per group). **b** qRT-PCR for the adipocyte specific gene *PLIN1* in mesothelial cells ± TGFβ1 and in a human adipocyte cell line (SGBS) before (D0) and after differentiation (D14)(n = 6 per group). **c** Expression of the epithelial cell marker *CDH*-*1* and the mesenchymal cell markers *VMAC*, *SNAI1, and SNAI2* before and after treatment of mesothelial cells with TGFβ1 (n = 6 per group)

We then wanted to determine if *ITLN1* expression requires an epithelial cell fate. Primary human mesothelial cells were treated with TGFβ1 (5 ng/ml) for 48 h in order to induce epithelial to mesenchymal transition. qRT-PCR demonstrated that *ITLN1* RNA expression was dramatically reduced within 48 h (Fig. 5a). We also confirmed that expression of *ITLN1* was absent in a human adipocyte cell line before and after adipogenic differentiation (SGBS D0 and D14); whereas the adipocyte specific gene Perilipin 1 (*PLIN1*) was increased dramatically in this cell line, but not in the mesothelial cells (Fig. 5b). Furthermore, using qRT-PCR, we confirmed that the decreased *ITLN1* mRNA expression was paralleled by an early transition to a mesenchymal lineage, characterized by increased expression of the mesenchymal markers *VMAC*, *SNAI1,* and *SNAI2* (Fig. 5c). Interestingly, many genes that have been shown to play a role in the epithelial to mesenchymal transition were among the complete list of RNA-seq differentially expressed genes from our pre- and post-ischemic human myocardial samples. In particular, there was upregulation of the mesenchymal markers *MMP9*, *TCF4*, *BMP1*, and *SNAI2*, and downregulation of the epithelial markers *KRT19* and *CDH*-*1* (Additional file 1: Table S1).

## Discussion

This is the first study to demonstrate changes in *ITLN1* expression in a large cohort of human left ventricular tissue samples following acute ischemia. We show that pre-ischemia levels of *ITLN1* expression in the human myocardium are much higher than the nominal levels depicted in the GTEX consortium [36]. This is likely a result of the tissue samples being immediately placed in preservative unlike other samples exposed to longer periods of inadvertent ischemic time prior to harvesting. The ischemic insult of CPB led to a dramatic decrease in *ITLN1* reads to very low detectable levels in most patients. Given results from prior studies which have shown a cardioprotective function of ITLN1 [5, 17], this decrease in endogenous *ITLN1* RNA may make the heart vulnerable to further injury. There was also a negative correlation between aortic cross clamp time and the level of post-ischemia *ITLN1* mRNA, although with the lack of significance we cannot conclude with certainty that longer ischemic time leads to lower levels of *ITLN1* mRNA in the myocardium.

Prior studies have shown that plasma levels of ITLN1 are low in patients with CAD [12, 19]. The plasma levels of ITLN1 prior to acute ischemia in our study were also lower in patients with known CAD, although this difference was not statistically significant. The response to acute ischemia has been less studied. One group showed that patients with acute coronary syndrome (ACS) have lower levels of plasma ITLN1 than healthy controls [13]. Our study using a large number of paired tissue samples, on the other hand, demonstrated an increase in plasma ITLN1 levels. This difference was significant regardless of underlying CAD. Those patients in the prior study with ACS likely had chronic levels of ischemia prior to presentation, and that may have been the driving force behind lower levels of ITLN1. As a result, chronic levels of low grade ischemia, as present in patients with CAD, may lead to low levels of plasma ITLN1, but acute ischemia may cause an abrupt increase. Future studies are needed to determine the source of this increased pool of circulating ITLN1. It could originate from the myocardium, which combined with lower levels of RNA expression would expose this organ to even more injury, or it could come from a visceral fat compartment in the body in order to rescue low myocardial expression. We did not identify a correlation between circulating levels of pre-ischemia ITLN1 protein and the myocardial injury marker CKMB on post-operative day 1. Nevertheless, the low variance in CKMB levels in our study does not refute other studies that have shown a potential protective effect of ITLN1 towards myocardial ischemia.

In agreement with previous work, we show that *ITLN1* is also expressed in EAT and localized to the SVF [7, 8, 37]. We further expand this knowledge by showing that ITLN1 is confined to the mesothelial cells of the visceral pericardium or epicardium in human tissue through immunohistochemistry. Recent studies have shown that the mesothelial layer plays critical roles in development and disease processes in fat tissue in part due to its ability to secrete regulatory substances in a paracrine fashion [38]. This localization is additionally supported by evidence that *ITLN1* expression is dependent on an epithelial cell fate as expression levels decrease following an epithelial to mesenchymal transformation. Interestingly, ischemic injury has been shown to stimulate an epithelial to mesenchymal transition in the epicardium, a process that can donate cells of multiple lineages to the myocardium [39, 40]. Many genes known to play a role in the epithelial to mesenchymal transition were dysregulated in our myocardial samples following ischemia. As a result, although it is possible that the changes in *ITLN1* expression may come from myocytes in our biopsies, it is also possible that these changes occur in a population of cells that can undergo an epithelial to mesenchymal transition following injury such as the vascular endothelium, the endocardium, the epicardium, or cells in transition from the endocardium/epicardium to the myocardium. Additional studies are required to further define this cell population.

## Conclusions

This is the first study to demonstrate an acute decline in left ventricular *ITLN1* expression following ischemia in a large cohort of surgical patients. This is paralleled by an increase in plasma protein levels. We also provide evidence to suggest that the expression of *ITLN1*, similar to other secreted factors in the heart, may be dependent on an epithelial cell fate. Future studies will be needed to validate the precise sources and targets of ITLN1 in the heart in order to further define its protective capabilities.

## Additional file

**Additional file 1: Table S1.** Differentially expressed genes affiliated with the epithelial to mesenchymal transition. The following table lists those genes associated with the epithelial to mesenchymal transition which are also among the list of significant differentially expressed genes following ischemia in human myocardial tissue biopsies with adjusted p-value <0.1 (Benjamini Hochberg method).

## Abbreviations

ITLN1: Omentin/Intelectin-1
VAT: visceral adipose tissue
SAT: subcutaneous adipose tissue
EAT: epicardial adipose tissue
SVF: stromal vascular fraction
CAD: coronary artery disease
CPB: cardiopulmonary bypass
FPKM: fragments per kilobase per million mapped reads
BMI: body mass index
HgA1c: hemoglobin A1c
CKMB: creatine kinase MB fraction
ACS: acute coronary syndrome
